# Mapping the Complex Transcriptional Landscape of the Phytopathogenic Bacterium Dickeya dadantii

**DOI:** 10.1128/mbio.00524-22

**Published:** 2022-05-02

**Authors:** Raphaël Forquet, Xuejiao Jiang, William Nasser, Florence Hommais, Sylvie Reverchon, Sam Meyer

**Affiliations:** a Université de Lyon, INSA-Lyon, Université Claude Bernard Lyon 1, CNRS UMR5240, Laboratoire de Microbiologie, Adaptation, Pathogénie, Villeurbanne, France; Universidad de Córdoba

**Keywords:** phytopathogen, transcriptional regulation, transcription unit, transcriptional read-through, transcription start and termination sites

## Abstract

Dickeya dadantii is a phytopathogenic bacterium that causes soft rot in a wide range of plant hosts worldwide and a model organism for studying virulence gene regulation. The present study provides a comprehensive and annotated transcriptomic map of *D. dadantii* obtained by a computational method combining five independent transcriptomic data sets: (i) paired-end RNA sequencing (RNA-seq) data for a precise reconstruction of the RNA landscape; (ii) DNA microarray data providing transcriptional responses to a broad variety of environmental conditions; (iii) long-read Nanopore native RNA-seq data for isoform-level transcriptome validation and determination of transcription termination sites; (iv) differential RNA sequencing (dRNA-seq) data for the precise mapping of transcription start sites; (v) *in planta* DNA microarray data for a comparison of gene expression profiles between *in vitro* experiments and the early stages of plant infection. Our results show that transcription units sometimes coincide with predicted operons but are generally longer, most of them comprising internal promoters and terminators that generate alternative transcripts of variable gene composition. We characterize the occurrence of transcriptional read-through at terminators, which might play a basal regulation role and explain the extent of transcription beyond the scale of operons. We finally highlight the presence of noncontiguous operons and excludons in the *D. dadantii* genome, novel genomic arrangements that might contribute to the basal coordination of transcription. The highlighted transcriptional organization may allow *D. dadantii* to finely adjust its gene expression program for a rapid adaptation to fast-changing environments.

## INTRODUCTION

Classically, bacterial transcription is described with the model of Jacob and Monod based on operons, defined as sets of contiguous and functionally related genes cotranscribed from a single promoter up to a single terminator (1). In recent years, however, accumulating studies have demonstrated that most operons actually comprise internal promoters and terminators, generating transcripts of variable gene composition, generally in a condition-dependent manner (2–5). This phenomenon, also known as suboperonic regulation (6), might be compared to alternative splicing in eukaryotes (7) and demonstrates a higher complexity of bacterial transcriptional landscapes than previously thought. Besides, transcription has been shown to extend beyond operons (3, 8), the latter being actually part of larger functional genomic units, referred to as transcription units (TUs) throughout this article.

While transcriptomic maps have been established for various bacteria, including Escherichia coli (9), Salmonella enterica (10), Bacillus subtilis (2), Streptococcus pneumoniae (4), Campylobacter jejuni (11), Clostridium beijerinckii (12), Mycobacterium tuberculosis (13), Mycoplasma pneumoniae (14), and the phytopathogen Xanthomonas campestris (15, 16), they are still lacking for *Dickeya* species. This study aims to provide a comprehensive and annotated transcriptomic map of Dickeya dadantii, a Gram-negative phytopathogenic bacterium representative of the *Dickeya* genus that causes soft rot, a severe disease leading to tissue maceration and eventually plant death (17) in a wide range of plant hosts worldwide, including agriculturally important crops (18–22).

The infection process involves an asymptomatic phase, in which bacteria remain latent and penetrate and colonize plant tissues, consuming simple sugars and small soluble oligosaccharides available in the plant apoplast to grow exponentially (23). In this compartment, bacteria are exposed to acidic conditions (24) and oxidative stress (25) resulting from plant defenses. When all nutrients are consumed in the apoplast, the symptomatic phase initiates. Bacteria produce plant cell wall-degrading enzymes (mainly pectinases) leading to the soft rot symptoms and start cleaving pectin, which is used as a secondary carbon source for a new round of growth (26). By causing a total destruction of plant cells, the maceration of plant tissues releases both vacuolar and cytoplasmic components in the apoplast, exposing the bacteria to osmotic stress (23).

In order to characterize the *D. dadantii* transcriptional landscape, we used a combination of transcriptomic data generated *in vitro* in a broad range of growth and stress conditions reflecting some of the key environmental signals encountered during the plant infection process and ensuring optimal reproducibility and quality of analyzed RNAs (27, 28). Different techniques were used that provided complementary knowledge: high-resolution Illumina paired-end RNA sequencing (RNA-seq), DNA microarray, Nanopore native RNA-seq, and differential RNA-seq (dRNA-seq). These data were combined using an integrative computational method developed for this study, allowing the inference of the RNA landscape and a validation of coexpression occurring among genes of the same TU. This analysis provides a detailed and annotated map of the TUs defining the *D. dadantii* transcriptome, i.e., the sets of contiguous coexpressed genes. We then quantitatively mapped transcription start and termination sites in the investigated conditions and analyzed the associated predicted promoter and terminator motifs. We show that TUs sometimes coincide with predicted operons but are generally longer, most of them exhibiting internal promoters and terminators. We characterized the occurrence of transcriptional read-through at terminators, a mechanism proposed as a basal coordinator and regulator of gene expression yet never explored in phytopathogens and still poorly understood across genomes in general. We finally detected putative noncontiguous operons and excludons in the *D. dadantii* genome. In order to validate the obtained transcriptional map, we analyzed available *in planta* expression data and show that TUs inferred from *in vitro* cultures are also coexpressed during the early stages of plant infection (29), suggesting that many of the analyzed features are used by *D. dadantii* in the pathogenic context. This transcriptomic map might serve as a community resource to help elucidate the regulation of *D. dadantii* gene expression, including its virulence program. It also provides insights into basal rules of coordination of transcription that might be valid for other bacteria, specifically for other *Dickeya* species for which a core genome of 1,300 genes has been identified by comparative genomics (30).

## RESULTS AND DISCUSSION

### Characterization of Dickeya dadantii transcription units.

**FIG 1:**
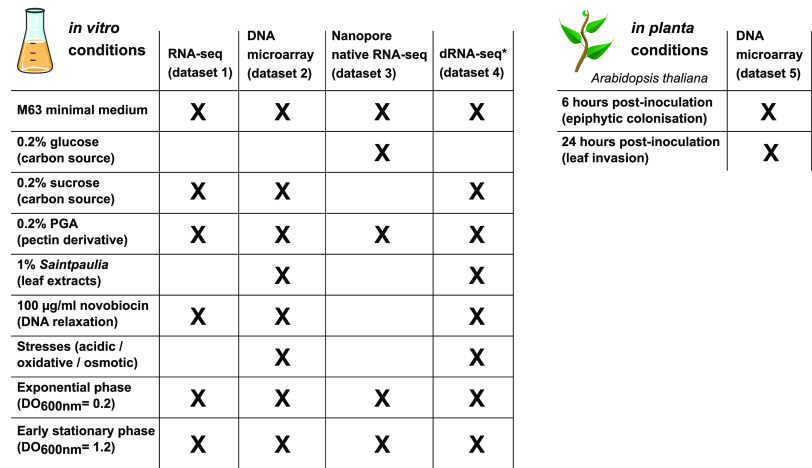
Graphical overview of the experimental approaches and growth conditions used for the mapping of the *D. dadantii* transcriptome. dRNA-seq*, RNAs from data set 2 were pooled into four samples.

In order to generate a biologically relevant transcriptional map of *D. dadantii*, we combined and integrated four sets of transcriptomic data obtained from *in vitro* cultures subjected to different sugar sources, environmental stress factors (acidic, oxidative, osmotic stress), and variations of DNA supercoiling (Fig. 1), reflecting a variety of conditions also encountered by bacteria in the course of plant infection. A fifth set obtained from bacteria grown *in planta* was used for validation (Fig. 1). These data were collected by different experimental methods providing complementary information, as follows (a more detailed description of the data sets is provided in Materials and Methods).

Data set 1 was generated from high-resolution Illumina paired-end, strand-specific RNA-seq covering six growth conditions: M63 minimal medium supplemented with sucrose, addition of polygalacturonate (PGA), a pectic polymer present in plant cell wall (31), and treatment by novobiocin, which induces a global and transient chromosomal DNA relaxation (32), in exponential or in early stationary phase (Fig. 1). By providing short but precise sequencing reads at single-base-pair resolution and high sequencing depth, this data set yields precise and quantitative information on the RNA landscape.

Data set 2 was generated from DNA microarray data covering 32 growth conditions, involving the presence of PGA and leaf extracts, and in each medium, a separate exposure to acidic, oxidative, or osmotic stresses (28) (Fig. 1). This data set provides a quantitative catalogue of genes’ responses to a more comprehensive and detailed range of conditions than data set 1, albeit of weaker spatial resolution.

Data set 3 was generated from long-read Nanopore native RNA-seq in M63 minimal medium supplemented with glucose and PGA, pooled from samples obtained in both exponential and early stationary phases (Fig. 1). This method allows native RNAs to be sequenced directly as near-full-length transcripts from the 3′ to 5′ direction, with a weaker depth than the previous data sets. Only a few transcriptomes were analyzed by this technique, mostly from viral and eukaryotic organisms (33–36) and, to our knowledge, a single prokaryotic one (37). This data set provides a direct isoform-level validation of the TUs and an accurate definition of transcription termination sites.

Data set 4 was generated from differential RNA sequencing (dRNA-seq) experiments carried out on four samples obtained by pooling RNAs from the large variety of environmental conditions of data set 2 (Fig. 1), followed by treatment with terminator exonuclease (TEX) prior to sequencing. TEX enzyme degrades processed 5′-monophosphate RNAs and consequently enriches the samples in primary 5′-triphosphate end transcripts (38), thus locating transcription start sites at single-nucleotide resolution.

Finally, data set 5 was generated from *in planta* DNA microarray data, 6 and 24 h postinoculation of the model plant Arabidopsis thaliana (29), during the early stages of infection (Fig. 1). Bacterial RNAs are difficult to isolate from plant tissues, especially during the symptomatic phase where phenolic compounds accumulate in decaying tissues, explaining the lack of transcriptomic data during the late stages of infection. In spite of a limited variety of conditions, this data set allows a comparison of gene expression profiles between *in vitro* and *in planta* experiments and was used to validate the level of coexpression of genes within TUs during the early stages of plant infection.

This collection of diverse and complementary transcriptomic data sets provided a solid ground for precisely characterizing the *D. dadantii* transcription units, rather than basing our analysis on genomic data alone as in most operon predictors (intergenic distances between genes, functional links among products). The employed algorithm is described in detail in Materials and Methods. Briefly, in a first step, we analyzed the RNA landscape from Illumina paired-end strand-specific RNA-seq (data set 1), ensuring good resolution and sufficient sequencing depth to obtain a quantitative signal for all genes. These data also allowed us to uncover 50 putative coding genes previously unannotated, most of which exhibit sequence homology with proteins from the *Dickeya* genus (see Table S1D in the supplemental material). Putative TUs were defined by fusing adjacent genes as long as RNA fragments were found in their intergenic region, a signature of cotranscription. Second, if genes within the same putative TU are indeed cotranscribed, they should exhibit strong correlation of expression under a wider range of conditions than those of data set 1. This analysis was carried out using the diversity of samples in our DNA microarray data (data set 2), based on a customized hierarchical clustering framework (39). This second criterion (correlation of expression) provided an orthogonal cross-validation compared to the first one (intergenic RNA signal) and yielded a total of 2,028 putative TUs along the *D. dadantii* genome. In a third step, these TUs were validated based on Nanopore native RNA-seq (data set 3). We tested the presence of long native RNA reads overlapping adjacent genes belonging to the same TU, thus yielding direct evidence of cotranscription. For 16% of adjacent gene pairs, no conclusion could be drawn because of insufficient coverage. Among the others, cotranscription was confirmed in 92% of the cases; for the remaining 8%, the absence of a common RNA might be indicative of false positives but, for some of them, may also be due to the low number of culture conditions included in data set 3. Since the large majority of TUs defined from data sets 1 and 2 match the observations of Nanopore native RNA-seq, we favored the latter hypothesis and retained all of them, with a confidence level reflecting the presence or absence of overlapping RNA reads (Table S1A).

10.1128/mbio.00524-22.6 TABLE S1 (A) Dickeya dadantii transcription units defined by our approach. (B) TSSs across TUs. (C) TTSs across TUs. (D) Unannotated protein coding genes. Download Table S1, XLSX file, 0.4 MB. Copyright © 2022 Forquet et al. 2022 Forquet et al. https://creativecommons.org/licenses/by/4.0/ This content is distributed under the terms of the Creative Commons Attribution 4.0 International license.

**FIG 2:**
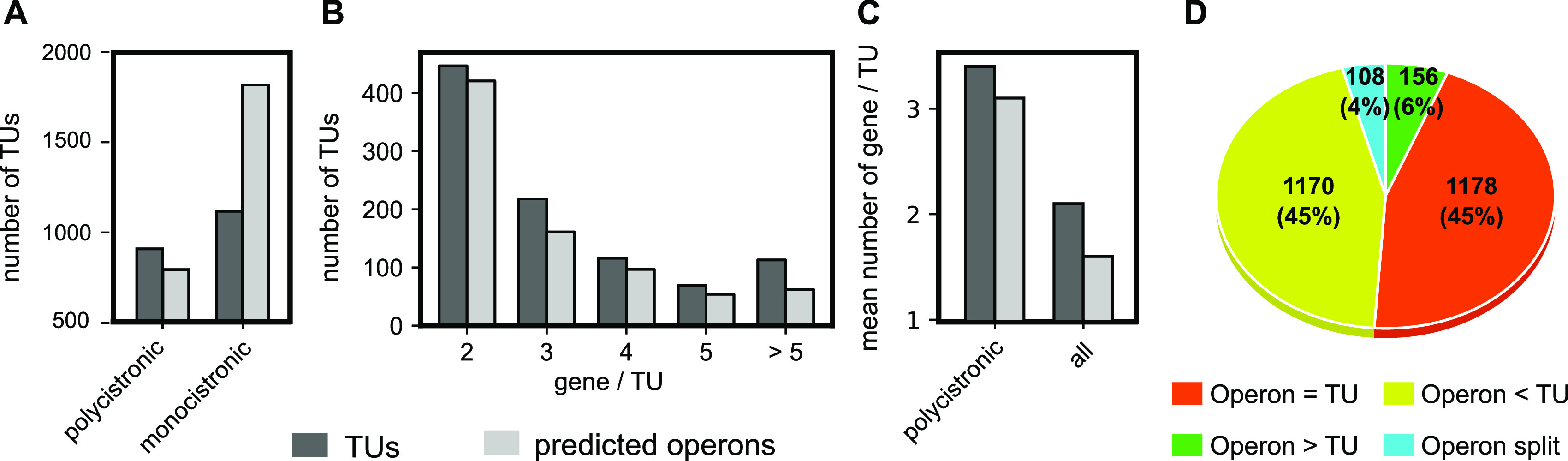
(A) Repartition of monocistronic and polycistronic TUs identified by our analysis and comparison to predicted operons. (B) Size distributions. (C) Average number of genes per TU. (D) Fate of predicted operons that are found mostly as or within TUs in our algorithm.

**FIG 3:**
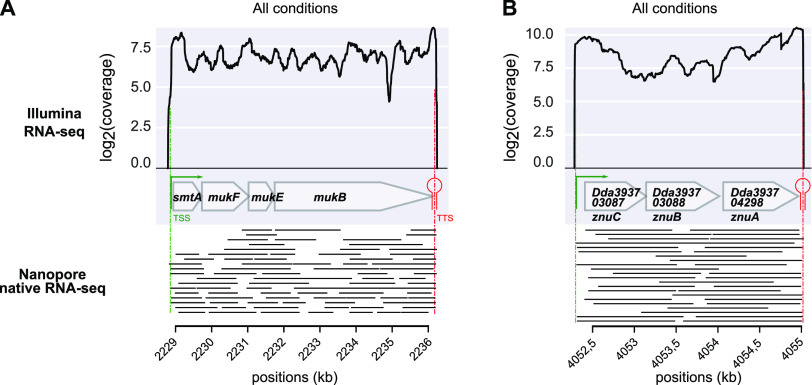
Transcription units identified by our approach and coinciding with operons. (A) Example of a known operon (*smtA-mukFEB*). The bottom panel shows the coordinates of the long native RNA reads sequenced by Nanopore. (B) Identification of a new TU exhibiting uniform read coverage and strong internal cross-correlations (Fig. S1A), clearly indicative of an operon. Its function was unknown, but a homology analysis revealed that it corresponds to the cluster of genes *znuCBA*, a Zn^2+^ uptake system (104). Long reads are observed for all adjacent gene pairs in Nanopore native RNA-seq data and even a fragment carrying the three genes for *znuCBA*.

With this approach, we mapped the first layer of transcription organization in *D. dadantii*. According to our findings, the 4,211 protein coding genes are organized into 2,028 transcription units (provided in Table S1A), among which 1,118 are monocistronic and 910 are polycistronic, ranging from 2 to 28 genes (Fig. 2A and B; Table S5). We carried out a functional enrichment analysis on the identified transcription units, using the gene ontology (GO) annotation system. We found that among the 910 polycistronic TUs, only 235 (26%) consist of genes sharing at least a common GO term. These TUs are significantly shorter (2.9 genes/TU on average) than the others (3.6 genes/TU on average; *t* test *P* value, <10^−6^). At the genomic scale, we compared our results with those of Rockhopper, a popular operon predictor that uses expression data as well as genomic information as input (40). Forty-five percent of predicted operons exactly coincide with a TU in our analysis (Fig. 2D), including known examples such as *smtA-mukFEB* involved in chromosome partitioning (Fig. 3A) (41). Besides, many identified TUs are likely operons of unknown functions and features (Fig. 3B), which represent interesting starting points to discover new transcriptional functional units. Remarkably, TUs are generally longer than predicted operons: the average TU (including monocistronic ones) contains 2.1 genes, and the average polycistronic TU contains 3.4 genes, compared to 1.6 and 3.1 genes, respectively, for predicted operons (Fig. 2C). Almost three-quarters (73.5%) of all genes are cotranscribed in TUs, against 56.9% for predicted operons (Fig. 2A; Table S5). Our results indicate that TUs are indeed larger functional genomic units, since 45% of predicted operons are extended by at least one gene (Fig. 2D), in agreement with recent findings in E. coli based on long-read sequencing (3).

10.1128/mbio.00524-22.10 TABLE S5 Catalogue of transcription unit architecture. Putative TUs are obtained from the first step of the approach (analysis of intergenic signal). Varying the precise value of the correlation threshold *C* for coexpression validation (step 2) does not change the results qualitatively. A larger *C* value results in shorter but more highly correlated TUs. Final TUs obtained with a *C *of 0.25 are longer than predicted operons and exhibit a length distribution similar to those reported in E. coli (3, 9). Download Table S5, XLSX file, 0.01 MB. Copyright © 2022 Forquet et al. 2022 Forquet et al. https://creativecommons.org/licenses/by/4.0/ This content is distributed under the terms of the Creative Commons Attribution 4.0 International license.

10.1128/mbio.00524-22.1 FIG S1 (A) Coexpression validation of *znuCBA* TU with *in vitro* DNA microarray data (data set 2): the three genes exhibit strong internal cross-correlations clearly indicative of an operon. (B) Coexpression validation of *sapABCDF-fabI* TU: the six genes are coexpressed, with a reduced correlation of *fabI* due to the presence of a strong internal TSS (Fig. 5A). (C) Coexpression validation of *rhlb-gppA-pehV* TU: the three genes are coexpressed, with a reduced transcriptional level of *pehV* (Fig. 6A) and a reduced correlation due to condition-independent read-through at the *gppA* intrinsic terminator. (D) Effect of PGA on *pelCZ* TU: *pelC* and *pelZ* expression profiles are similar in the absence (left) or presence (right) of PGA in stationary phase, in spite of a drastically different global expression level. Download FIG S1, PDF file, 0.10 MB. Copyright © 2022 Forquet et al. 2022 Forquet et al. https://creativecommons.org/licenses/by/4.0/ This content is distributed under the terms of the Creative Commons Attribution 4.0 International license.

As an example, the *sapABCDF* operon encoding a transporter involved in antimicrobial peptide resistance and virulence in numerous bacteria, including *D. dadantii* (42), is extended to include the enoyl-acyl carrier protein reductase gene *fabI* that catalyzes an essential step in the biosynthesis of fatty acids of the membrane (43) (see Fig. 5A). It might be noted that *fabI* has a different genomic location in E. coli and is consequently not cotranscribed with *sapABCDF* in that species (44), although this synteny is conserved in other *Dickeya* genomes, showing that TUs can merge and/or vary over time at the evolutionary scale. Since these genes are functionally unrelated (except for a general relation with the membrane), the biological relevance and putative role of this event require further investigation.

The *glg* genes involved in glycogen metabolism constitute another instructive example. They were initially classified in two separate operons in E. coli (45) and later identified as a single TU involving alternative transcripts of variable gene composition depending on growth conditions (46). The latter is also true in *D. dadantii* according to our findings (see Fig. 5C), illustrating how transcription extends beyond the scale of the operon.

### Genome-wide identification of *D. dadantii* transcription start and termination sites.

Once *D. dadantii* transcription units were defined, the next step was to elaborate a map of transcription start sites (TSSs) and transcription termination sites (TTSs) for each TU along the genome. First, dRNA-seq experiments were carried out (38) to build a large library of 9,288 putative TSSs at high resolution, covering a wide range of *in vitro* cultures under conditions also encountered during plant infection (data set 4) (Fig. 1; Table S2A). These were obtained by treating the RNA samples with TEX prior to sequencing, and the TSSer workflow was applied for a precise determination of TSS positions (47), followed by visual curation and classification (Fig. S3A). For TTSs, two sets of putative positions were generated based on (i) Nanopore native RNA-seq (data set 3), in which transcripts are sequenced from the 3′ ends, allowing the detection of 1,165 TTS positions based on the enrichment of these ends downstream of gene stops (Table S2D), and (ii) genome-wide predictions of termination sites, based on the two main mechanisms of transcription termination in bacteria. A total of 3,564 rho-independent (intrinsic) TTSs and 5,851 rho-dependent (regulated) TTSs (48) were predicted using ARNold (49) and RhoTermPredict (50) programs, respectively (Table S2B and S2C).

10.1128/mbio.00524-22.3 FIG S3 (A) Classification of TSSs identified by dRNA-seq (data set 4) depending on genomic location. (B) Quantification of condition-dependent transcriptional read-through: example of the *cytABCD* TU. A putative rho-independent TTS is identified downstream of *cytA* but not validated. Its probability of termination [inferred from the expression variation Δ(log_2_(RPKM)) of *cytA* compared to the other genes] is regulated and depends both on the growth phase and the presence of PGA [*P*(TTS*_cytA_*) = 0.78 ± 0.03 versus 0.51 ± 0.03] besides a global up-regulation of the whole TU. (C) The *greB* and *ompR-envZ* transcription units form a potential divergent excludon: long 5′ UTRs overlapping transcripts are generated by *ompR* and *greB* divergent genes and might form a dsRNA that could prevent each other’s transcription other. In E. coli, *ompR* and *envZ* are part of the same operon (red), and *greB* is transcribed alone (blue). Such a genomic region forming a dsRNA was also identified in E. coli (91). Download FIG S3, PDF file, 0.1 MB. Copyright © 2022 Forquet et al. 2022 Forquet et al. https://creativecommons.org/licenses/by/4.0/ This content is distributed under the terms of the Creative Commons Attribution 4.0 International license.

10.1128/mbio.00524-22.7 TABLE S2 (A) Putative TSSs identified by differential RNA-seq (TEX treatment) under a wide range of environmental conditions. (B) Genomic position and secondary structure of putative TTSs: intrinsic terminators predicted by ARNold (Erpin and RNAmotif algorithms). (C) Genomic position of putative TTSs: rho-dependent terminators predicted by RhoTermPredict. (D) Putative TTSs identified by Nanopore native RNA-seq. Download Table S2, XLSX file, 0.6 MB. Copyright © 2022 Forquet et al. 2022 Forquet et al. https://creativecommons.org/licenses/by/4.0/ This content is distributed under the terms of the Creative Commons Attribution 4.0 International license.

**FIG 4:**
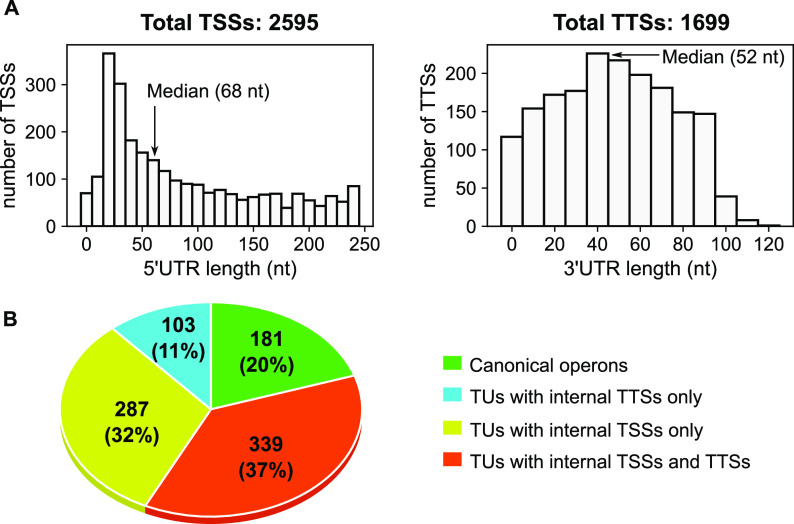
Global features of the *D. dadantii* transcriptome. (A) Distribution of 5′-UTR (left panel) and 3′-UTR (right panel) lengths among TSSs and TTSs. (B) Quantitative mapping of TSSs and TTSs across TUs.

A quantitative mapping of the transcription landscape was then performed in order to estimate the contribution of each TSS/TTS to its TU. While most comparable maps define TSSs/TTSs by their position only, we exploited the complementarity of the input data to also systematically analyze their magnitude (or strength) under the investigated conditions. The +TEX libraries, Nanopore reads, and TTS predictions are not suitable for the latter purpose, which required building a second list of TSSs and TTSs of poorer resolution but quantitative magnitude from the nontreated paired-end RNA-seq data (data set 1). Briefly, TSSs and TTSs were defined based on the enrichment in RNA fragment starts and stops upstream of gene starts and downstream of gene stops, respectively, and the number of fragments associated with these sites across all samples was considered the global strength. The lists obtained by the three methods (from data sets 1, 3, and 4) were then merged into a unified list of TSSs/TTSs of optimal spatial resolution, quantitative magnitude, and with an estimated level of confidence depending on the level of agreement between these data sets (see Materials and Methods). These TSSs and TTSs were then assigned to the TUs. In order to eliminate many very weak internal TTSs/TTSs (most of which likely have poor biological relevance), the latter were retained only if they yielded at least 15% of the total start/stop magnitude of the TU and were thus used at least in some of the investigated conditions. As a result, we defined a total of 2,595 TSSs and 1,699 TTSs (including internal ones) over all TUs (Fig. 4; Table S1A to S1C). Inevitably, some alternate TSSs/TTSs may be absent from these lists if they are specifically used under conditions not included in our data sets. 5′ and 3′ untranslated regions (UTRs) exhibit median lengths of 68 and 52 nucleotides (nt), respectively (Fig. 4A). Finally, a scan for promoter motifs, conducted with bTSSfinder (51), identified promoters upstream of 1,848 (71%) TSSs in total (Table S1B); the absence of detected promoters for the remaining 29% TSSs was expected due to the limitations of such predictors (52). In addition, we manually annotated HrpL predicted promoters based on previous analysis in *D. dadantii* (53) and various phytopathogenic bacteria (54) (Table S1B). HrpL is an alternative sigma factor that binds to the hrp box promoter sequence of genes involved in the type III secretion system (53, 54). To evaluate the quality of our TSS definition, we compiled all experimentally determined TSSs in *D. dadantii* (by primer extension) and compared their positions to our findings (Table S3). Forty-five percent displayed exactly the same position, 38% were distant by less than 5 nucleotides, and only 17% were distant by more than 6 nucleotides. Manually annotated promoter elements from these studies also match our findings well (Table S3).

10.1128/mbio.00524-22.8 TABLE S3 TSS validation, based on all published TSSs to date and to our knowledge in *D. dadantii*. Download Table S3, XLSX file, 0.01 MB. Copyright © 2022 Forquet et al. 2022 Forquet et al. https://creativecommons.org/licenses/by/4.0/ This content is distributed under the terms of the Creative Commons Attribution 4.0 International license.

### Characterization of a complex transcriptional landscape.

**FIG 5:**
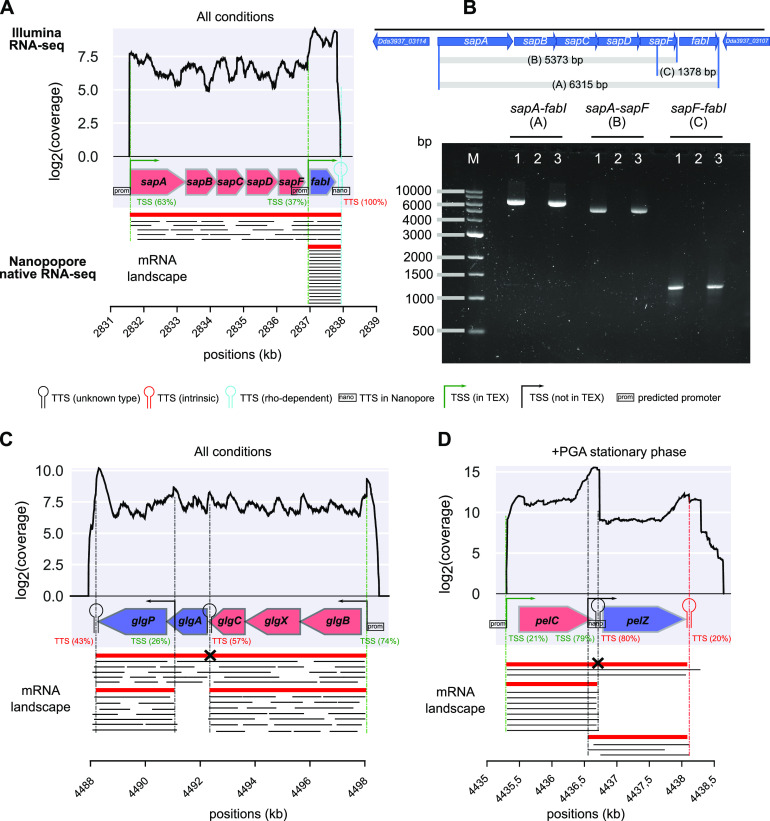
(A) The *sapABCDF* and *fabI* genes, predicted by Rockhopper (40) as two separate (red and blue) operons, were identified as a single TU, with a strong internal TSS expressing *fabI* alone. The bottom panel indicates the different isoforms (red) and the long reads sequenced by Nanopore native RNA-seq (black). The latter overlap all adjacent gene pairs, providing direct evidence for cotranscription. (B) Transcriptional organization of the *sapABCDF-fabI* locus analyzed by RT-PCR, using RNAs extracted from bacteria grown in M63 medium supplemented with sucrose, in exponential phase (optical density at 600 nm, 0.2). For each pair of primers, three reactions were performed using RT-PCR kits from TaKaRa: (i) the RT-PCR assay, (ii) a negative control without reverse transcriptase, and (iii) a positive control using genomic DNA instead of cDNA. Lane M corresponds to a 1-kb DNA ladder from NEB. (C) The *glg* genes were identified as a single TU (involving several isoforms) containing two separate predicted operons (blue and red genes), as suggested by the uniform read coverage, long reads from Nanopore native RNA-seq (bottom), and in line with results in E. coli (3, 46, 75). (D) Identification of the *pelCZ* TU with different isoforms depending on the condition, as previously determined (56). The two genes are split into different operons by Rockhopper. A strong internal TSS, followed by a strong TTS, contributes to the complexity of its expression (see the text). Long reads corresponding to the different mRNA isoforms (*pelC*, *pelZ*, or *pelCZ*) are observed.

**FIG 6:**
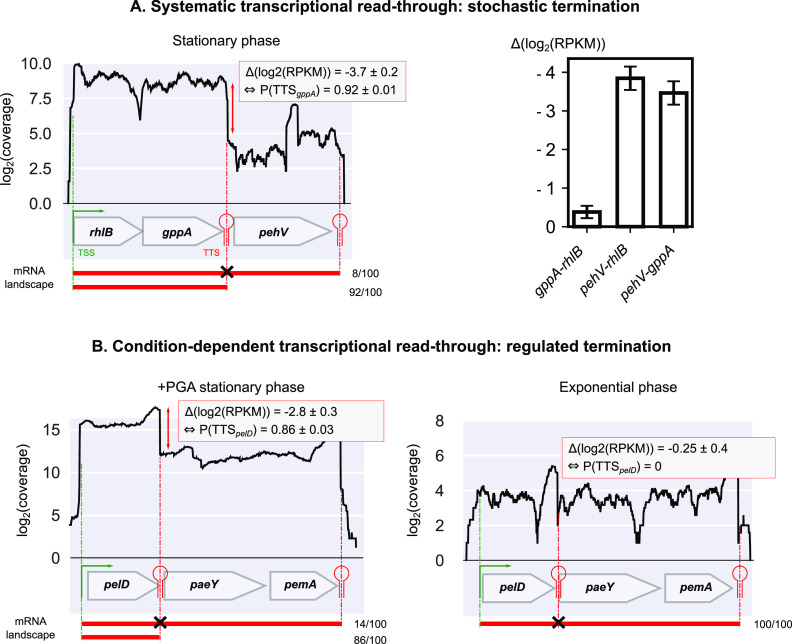
Quantification of transcriptional read-through. (A) Nonconditional read-through: example of the *rhlB*-*gppA*-*pehV* TU (left panel). The first two genes are homogeneously transcribed among conditions, resulting in an expression variation of Δ(log_2_(RPKM)) close to 0 (right panel; 95% confidence intervals are shown), while the intrinsic TTS downstream of *gppA* is stochastically overstepped in 8% ± 1% of transcripts [*P*(TSS*_gppA_*) = 0.92 ± 0.01], resulting in two different isoforms (red). (B) Condition-dependent read-through: example of the *pelD*-*paeY* -*pemA* TU. A TTS is identified downstream of *pelD* in agreement with previous studies (78). Its termination probability is regulated and depends on growth phase and presence of PGA (0.86 ± 0.03 versus 0), besides a global up- or downregulation of the whole TU. All mRNA isoforms are observed in Nanopore native RNA-seq data (Fig. S2A and S2B).

**FIG 7:**
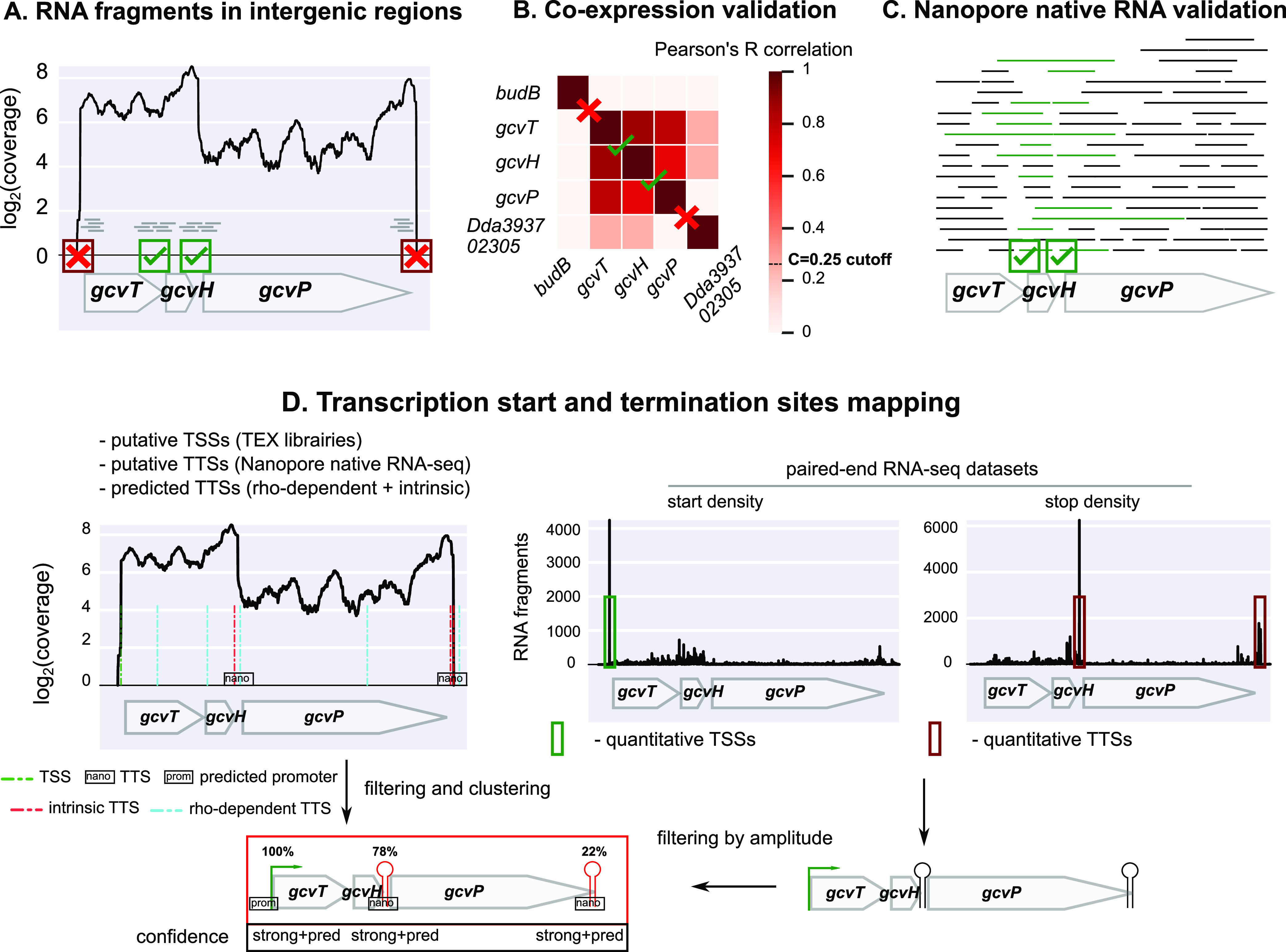
Algorithm for the characterization of *D. dadantii* transcription units. (A) Definition of putative TUs based on RNA-seq coverage (data set 1) in intergenic regions, with isodirectional genes being split when the coverage drops to zero (here, before *gcvT* and after *gcvP*). (B) Validation based on correlation of expression across 32 conditions (data set 2). The genes of identified putative TUs are correlated, in contrast to surrounding isodirectional genes (*budB* and *Dda3937_02305*). (C) Validation based on Nanopore native RNA-seq, based on the presence of overlapping RNA reads between adjacent gene pairs, yielding direct evidence for cotranscription. (D) TSS and TTS mapping based on dRNA-seq (TEX libraries; data set 4), Nanopore native RNA-seq (data set 3), TTS predictions, promoter predictions, and paired-end RNA-seq data (data set 1). First, putative TSSs and TTSs of high resolution but qualitative strength were defined from an analysis of TEX libraries and Nanopore native RNA-seq, respectively, and rho-dependent/intrinsic terminations were predicted. Second, a list of TSSs and TTSs of quantitative strength but poorer resolution was defined from the enrichment of RNA-seq paired-end fragment starts (start density) and stops (stop density) upstream of gene starts and downstream of gene stops, respectively. Third, only TSSs and TTSs with sufficient strength were retained and compared to the closest TEX TSS/Nanopore TTS/predicted hairpin loop, in order to define their exact position and level of confidence. Finally, promoters were predicted for the retained TSSs. As a result of the analysis, this TU included the *gcvTHP* genes, the first two genes being expressed both as part of the entire transcript and as an independent transcript generated from a strong internal TTS (76% of total magnitude), explaining the lower correlation between *gcvP* and the remaining genes.

The quantitative mapping of TSSs and TTSs allowed us to refine the comparison of TUs and operons presented above. According to our findings, only 20% of polycistronic TUs (181) exhibit a single promoter and terminator (Fig. 3 and 4B) and thus fit into the classical definition of operons, and only 47% of these (85) are predicted as such by Rockhopper. The 80% remaining TUs (729) are complex (Fig. 4B). Thirty-two percent (287) have at least one internal TSS without any internal TTS, such as *sapABCDF-fabI* (Fig. 5A and B). Thirty-seven percent (339) have both internal TSS(s) and TTS(s), such as *glgBXCAP* (Fig. 5C) and *pelCZ* (Fig. 5D). Finally, 11% (103) have at least one internal TTS without any internal TSS, such as *rhlB*-*gppA*-*pehV* (Fig. 6A), *pelD*-*paeY* -*pemA* (Fig. 6B), and *gcvTHP* (Fig. 7). Most *D. dadantii* TUs can consequently generate alternative transcripts of variable gene composition, resulting in a dense and complex transcriptional landscape.

10.1128/mbio.00524-22.2 FIG S2 Cotranscription and mRNA landscape validation with Nanopore native RNA-seq for *rhlB-gppA-pehV* TU with condition-independent read-through at the intrinsic TTS downstream of *gppA* (A), *pelD-paeY-pemA* TU with condition-dependent read-through downstream of *pelD* internal TTS (B), and *cytABCD* TU with condition-dependent read-through downstream of *cytA* internal TTS (C). Those internal TTSs are occasionally overstepped, resulting in different transcripts isoforms (as shown in red), which are all detected as long native RNA reads (black). (D) *indC-vfmAZBCDFG* noncontiguous TU (black Nanopore reads on the negative strand), with *vfmE* being transcribed on the opposite strand (blue Nanopore reads on the positive strand), resulting in overlapping antisense transcripts. Download FIG S2, PDF file, 0.2 MB. Copyright © 2022 Forquet et al. 2022 Forquet et al. https://creativecommons.org/licenses/by/4.0/ This content is distributed under the terms of the Creative Commons Attribution 4.0 International license.

A notable feature of complex TUs is the heterogeneity of transcription levels along the genes due to internal TSSs/TTSs, usually in a condition-dependent manner, resulting in a moderate correlation in the expression of genes within the TU (9). As an example, in the *sapABCDF-fabI* TU (Fig. 5A), *fabI* is expressed both as part of the entire transcript, as validated by reverse transcription (RT)-PCR (Fig. 5B), and as an independent transcript generated from a strong internal TSS (Fig. 5A). In *glgBXCAP* (Fig. 5C), alternative transcripts of variable gene composition can be generated depending on TSS and TTS usage. Another example relevant to plant infection is the *pelCZ* cluster (Fig. 5D) encoding two endopectate lyases secreted by *D. dadantii* which degrade pectin contained in plant cell walls (55). The substrates of Pel enzymes are pectic oligomers, e.g., PGA, that act as inducers of *pel* expression (31). The *pelCZ* genes were previously shown by Northern blotting to be cotranscribed into a single polycistronic transcript under inducing conditions by PGA, in addition to the two monocistronic mRNAs encoded by *pelC* or *pelZ* under noninducing conditions (56). Our present findings are in full agreement with these observations, as *pelCZ* is detected as a single TU harboring one internal TSS and one internal TTS, each giving rise to monocistronic transcripts. In our data, *pelCZ* expression profiles are similar in the presence or absence of PGA in spite of a drastically different global expression level (Fig. S1D), suggesting that in the absence of inducer, the very low level previously prevented a reliable detection of the entire transcript. Together, our findings clearly indicate that the canonical operon model is insufficient to explain the complexity of the *D. dadantii* transcriptional landscape, in line with results in many other organisms (2–5). The existence of alternative entry and exit points for RNA polymerase inside TUs allows the cells to adjust the relative expression level of adjacent genes within a global coordination of expression of the entire TU (Fig. 5), which may allow, in the case of *D. dadantii* during plant infection, a rapid adaptation to changing environment.

### Transcriptional read-through, the root of transcription extension?

We showed that transcription units comprise predicted operons yet are generally longer. This extension of transcription might, in part, result from the ability of RNA polymerase to stochastically override an imperfect terminator by a mechanism referred to as transcriptional read-through (3, 8). The latter has long been identified in specific operons (57–59) and was shown more recently to be widespread in bacterial genomes (2, 3, 8), where it may in fact play a basal coordination and regulation role (5). A condition-independent rate of stochastic termination might result in the coexpression of the genes located before and after the TTS (as in a classical operon) but with a reduced transcriptional level of the latter, a mechanism possibly relevant to functionally related genes that must be expressed at different strengths while keeping a constant ratio (59). The termination efficiency can also be subject to regulation, depending on environmental conditions and metabolic needs, resulting in a variable degree of read-through and thus of relative expression levels (57, 58). Such conditional read-through can involve rho and other proteins assisting termination (60–63) as well as other conditional premature termination mechanisms such as attenuation (64, 65), T-box conditional termination (66, 67), and riboswitches (68, 69).

An example of condition-independent read-through occurs at the *rhlB*-*gppA*-*pehV* TU (Fig. 6A; Fig. S1C). The *rhlB* gene encodes a component of the RNA degradosome (70, 71), whereas *gppA* encodes GTP 3′-diphosphate (pppGpp) pyrophosphatase, involved in the bacterial stringent response (72), and the *pehV* gene encodes a polygalacturonase involved in pectin degradation (73). These genes are functionally unrelated (except for a distant link to nutritional stress) yet appear cotranscribed, which is in fact quite frequent among operons (41, 74). This TU exhibits a variable expression level (by up to 50%) across the sampled conditions, but the internal (relative) expression pattern is condition independent: *rhlB* and *gppA* are expressed at a similar level, whereas *pehV* is systematically less transcribed (Fig. 6A; Fig. S1C). This observation is correlated with the presence of an intrinsic internal TTS downstream of *gppA*. By computing the expression ratio of *pehV* to *rhlB* and *gppA*, we inferred the associated termination probability (or terminator strength) and found a constant value *P*(TTS*_gppA_*) of 92% ± 1% (95% confidence interval) characteristic of a nonconditional transcriptional read-through. Thus, the three genes are cotranscribed from a single promoter of condition-dependent activity, with a reduced transcriptional level of *pehV* exhibiting a constant ratio (8%) compared to the other genes. The biological relevance of this mechanism remains to be clarified. In E. coli, *rhlB* and *gppA* were also recently shown to be cotranscribed (3, 75). Another example of condition-independent read-through occurs at the *gcvTHP* TU involved in glycine cleavage (76) (Fig. 7). We detected an internal TTS downstream of *gcvH* in accordance with studies in E. coli (3, 75) and inferred a termination probability, *P*(TTS*_gcvH_*), of 71% ± 22% (95% confidence interval), based on the expression ratio of *gcvP* to *gcvT* and *gcvH* across RNA-seq conditions. It is unclear whether this variability is due to RNA-seq signal variations or a weak regulation of the termination rate. The GcvT, GcvH, and GcvP proteins are part of the glycine cleavage system with GcvL (77), and GcvP activity might be required at a lower concentration under the investigated conditions.

By definition, all identified internal TTSs (549) experience transcriptional read-through. As a rough estimate, condition-independent read-through was detected for 77 (14%) of internal TTSs, based on the constant expression ratio of the genes located downstream versus upstream across RNA-seq conditions (Fig. 6) (see Materials and Methods). The remaining internal TTSs rather experience condition-dependent read-through; however, the systematic estimation of stochastic termination rates at internal TTSs is delicate based on our data only, due to the limited number of RNA-seq conditions and the presence of nearby TSSs that contribute to the heterogeneous expression levels along the TU, as illustrated by *pelCZ* (Fig. 5D).

An example of condition-dependent read-through occurs at the *pelD*-*paeY*-*pemA* TU (Fig. 6B; Fig. S2B), which is identified by our approach but was also characterized by Northern blotting (78). It contains three genes involved in pectin degradation. In the initial step of pectinolysis occurring in plants, PaeY (acetylesterase) and PemA (methylesterase) remove acetyl and methyl groups from pectin, which can then be efficiently degraded by the pectate lyase PelD (17). The *pelD* gene is essentially transcribed as a monocistronic RNA, although its terminator (predicted as intrinsic) can be overstepped to generate a polycistronic transcript comprising the three genes (78). In exponential phase, the three genes are homogeneously (but weakly) transcribed as a unique polycistronic RNA, suggesting that the internal TTS is not efficient [*P*(TTS*_pelD_*) = 0%]. In stationary phase in the presence of PGA, the whole TU is upregulated, and the internal TTS becomes more efficient [*P*(TTS*_pelD_*) = 86% ± 3%, 95% confidence interval], resulting in the extensive synthesis of the *pelD* monocistronic RNA and a lower expression level of the two downstream genes. The regulation events occurring at this TTS remain to be characterized but may adjust the relative expression levels of the genes in accordance with metabolic needs, since PelD has a predominant role in pectin degradation and virulence (79, 80) and must likely be required at much higher concentrations than the two other enzymes. In addition, the fact that *pemA* is differentially expressed depending on the degree of pectin methylation (81) highlights the relevance of adjusting the relative expression levels of the three genes depending on plant cell wall composition.

Another example occurs at the *cytABCD* TU (Fig. S2C and S3B). In addition to plants, *D. dadantii* is able to infect insects (82), during which this TU expresses four insecticidal toxins and was previously shown to produce a polycistronic mRNA comprising the four genes, besides the possible existence of alternative isoforms (83). The sequencing coverage together with the putative internal intrinsic TTS detected after *cytA* are clearly indicative of a condition-dependent read-through, with termination occurring less efficiently at *cytA* in stationary phase in the presence of PGA than in exponential phase. This variation in termination efficiency at *cytA* associated with an environmental change may again allow tuning of the relative amounts of the corresponding toxins, especially if a precise and condition-dependent balance between them is required for optimal activity during the insect infection process (83). Interestingly, this cluster of four genes was acquired by horizontal transfer. Since transcriptional read-through partly relies on basal RNA polymerase-TTS interactions, it might be conserved during horizontal transfer among bacterial species without requiring an independent acquisition of regulatory signals and their integration in the transcriptional regulatory network of the recipient cell.

### Detection of putative excludons and noncontiguous transcriptions units.

**FIG 8:**
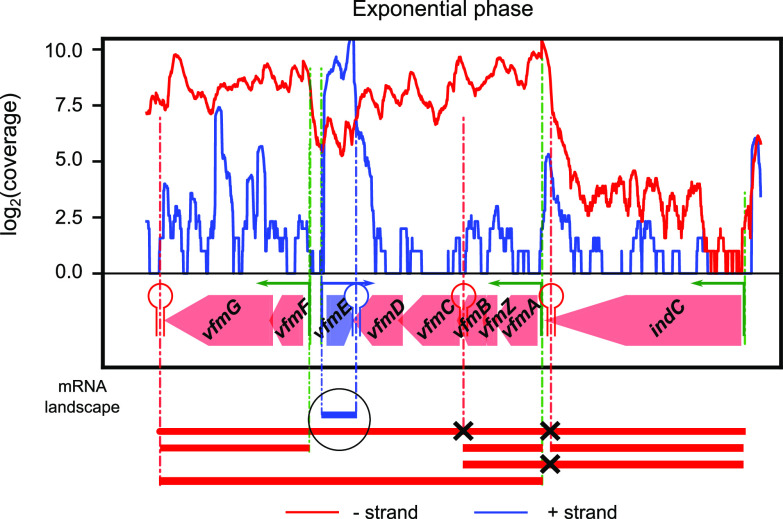
Existence of a potential noncontiguous transcription unit in the *vfm* locus. The *vfmE* gene is transcribed in the opposite direction of the *indC-vfmAZBCDFG* TU, generating two overlapping mRNAs (as shown in red/blue for the − or + strand) that might be involved in a mutual regulation (see the text). All mRNA isoforms are observed in Nanopore native RNA-seq data (Fig. S2D), including a long native RNA read on the negative strand between *vfmD* and *vfmF*.

All previous examples involved genes located on the same DNA strand, yet recent studies also describe interactions between overlapping antisense coding transcripts involved in a mutual regulation. In particular, noncontiguous operons refer to operons that contain a gene or group of genes that is transcribed in the opposite direction (84). Eighty-three TUs with such features were found in the *D. dadantii* genome (provided in Table S4A). Among them, an example is the *indC-vfmAZBCDFG* TU encoding a component of the *vfm* quorum sensing system required for the production of plant cell wall-degrading enzymes (Fig. 8; Fig. S2D) (85). The *vfmE* gene, located on the opposite strand and within this TU, is also part of this system and known to encode a transcriptional activator of the *vfm* locus (of the AraC family). Since all genes of the TU are cotranscribed within a single mRNA, it is likely that these two overlapping antisense transcripts could negatively regulate each other, e.g., by transcriptional interference (RNA polymerase collision) or RNase III-mediated double-stranded RNA (dsRNA) processing (86). An expression increase of the *vfm* locus would then reduce the expression of *vfmE* and, in turn, its own expression, forming a genome-embedded negative feedback loop controlling the production of quorum sensing signal and plant cell wall-degrading enzymes (87).

10.1128/mbio.00524-22.9 TABLE S4 (A) Catalogue of putative noncontiguous transcription units. (B) Catalogue of putative excludons. Download Table S4, XLSX file, 0.01 MB. Copyright © 2022 Forquet et al. 2022 Forquet et al. https://creativecommons.org/licenses/by/4.0/ This content is distributed under the terms of the Creative Commons Attribution 4.0 International license.

Finally, “excludons” refers to genomic regions in which convergent or divergent genes display overlapping transcription (88). From the map of transcription start and termination sites, we found 160 putative convergent excludons (overlapping 3′ UTRs) and 63 putative divergent excludons (overlapping 5′ UTRs) (provided in Table S4B). An example is the divergent excludon between *greB* and *ompR-envZ* transcription units, which encode a transcript cleavage factor required for effective transcription elongation (89) and a two-component signal transduction system involved in osmotic stress response (90), respectively (Fig. S3C). Both TUs comprise long 5′ UTRs, forming a region of overlapping transcription that was previously identified in E. coli (91) and might underpin a mutual posttranscriptional regulation.

### *In planta* coexpression validation of the transcription units.

While our transcriptional map was inferred from *in vitro* cultures, where RNAs could be extracted with optimal quality and reproducibility, we wished to test if the identified TUs could play a role under conditions of plant infection. We analyzed a set of expression data obtained *in planta* by DNA microarrays during the early stages of Arabidopsis thaliana infection (data set 5) (29), 6 h postinoculation, during the epiphytic colonization of leaf surface, and 24 h postinoculation during leaf invasion, just before the onset of visible symptoms. Overall, among the 50% gene pairs most correlated *in planta*, 80% belong to the same TUs, suggesting that cotranscription of these genes may indeed likely occur under these conditions (Fig. S4A). As an example, in *cytABCD*, the four genes are also highly correlated *in planta*, while this correlation immediately drops in surrounding isodirectional TUs (Fig. S4B), as we expected. However, comparable correlations might also arise between other genes that are not transcribed together but share the same transcriptional regulators, in particular those involved in virulence, such as KdgR, PecT, and PecS (92), thus accounting for the 20% strongly correlated gene pairs not located in the same TU. For example, in the *pelCZ* complex TU involved in pectinolysis, both genes are strongly correlated *in planta* (Fig. S4C), as expected from the previous *in vitro* observations (especially with PGA) (Fig. S4C), but the adjacent *pelB* gene is also correlated, whereas *crp* and *mrcA* are not. This is not surprising, since most *pel* genes are paralogous genes with similar regulators and are strongly induced by pectin. The same pattern is observed for the *pelD-paeY-pemA* TU (Fig. S4D), with respect to the *pelE* and *pelA* genes located upstream on the same strand. Because of the limited spatial resolution of microarrays and the low number of investigated conditions, it is not possible to systematically distinguish the effects of these two mechanisms at the genomic scale from these data, but a survey of representative TUs confirmed that they usually coincide with correlated blocks of genes (as observed with *cytABCD*), even when the latter do not belong to the same functional pathways.

10.1128/mbio.00524-22.4 FIG S4 *In planta* DNA microarray data, 6 h postinoculation (hpi) of the plant Arabidopsis thaliana (epiphytic colonization of the leaf surfaces, 5 replicates) and 24 hpi (leaf invasion, 4 replicates). (A) Distribution of coexpression correlation coefficients among all genes (blue) and among genes belonging to the same TU (red). Among the 50% most correlated genes *in planta*, 80% belong to the same TUs, with the example TUs from the present study (*smtA-mukFEB*, *znuCBA*, *sapABCDF-fabI*, *glgBXCAP*, *pelCZ*, *rhlB-gppA-pehV, pelD-paeY-pemA*, *cytABCD*) displaying a median correlation of 0.9 *in planta*. (B to D) Pearson’s coexpression correlation coefficients of *cytABCD* TU (B), *pelCZ* TU (C), and *pelD-paeY-pemA* TU (D) with surrounding isodirectional (on the same strand) TUs. (E) Identification of *sufABCDSE-ldtC* complex TU, composed of operons of apparently unrelated functions, exhibiting a strong internal TSS (51% total magnitude) upstream of *ldtC* (previously *ycfS*) and a strong internal TTS (52% total magnitude) downstream of *sufE*, allowing separate transcriptions. The seven genes are highly correlated *in planta*. Download FIG S4, PDF file, 0.6 MB. Copyright © 2022 Forquet et al. 2022 Forquet et al. https://creativecommons.org/licenses/by/4.0/ This content is distributed under the terms of the Creative Commons Attribution 4.0 International license.

As an example, the complex TU *sufABCDSE-ldtC* is composed of two functionally unrelated operons (Fig. S4E). *sufABCDSE* encodes components of the iron-sulfur cluster assembly machinery (93), which is required to synthesize and repair damaged iron-sulfur clusters under conditions of oxidative stress or iron limitation, and is therefore critical for *D. dadantii* virulence (92). In contrast, *ldtC* (previously *ycfS*), encodes a l,d-transpeptidase crucial for bacterial envelope assembly, by catalyzing the attachment of the major outer membrane protein Lpp to peptidoglycan (94). According to our findings above, *sufABCDSE* and *ldtC* can be transcribed together, with an internal TTS and TSS located between them. *In planta*, the seven genes are indeed strongly coexpressed, with a slight decrease for *ldtC*, in full agreement with the identified transcriptional map (Fig. S4E). It is conceivable that these genes are required under a common set of conditions encountered during plant infection, which was favored by their inclusion in the same transcript, while the presence of alternative TSS and TTS might still allow separate expression when required. Indeed, the *sufABCDSE* operon is controlled by three transcriptional regulators, Fur, OxyR, and IscR, which sense iron limitation, oxidative stress, and intracellular iron-sulfur cluster status, respectively (95). Each of them contributes to the activation of the *suf* promoter by oxidative stress occurring during plant penetration and colonization (25): the repressor Fur is inactivated by reactive oxygen species (ROS), the activator OxyR becomes active through the oxidation of two cysteine residues and the formation of a disulfide bond, and IscR becomes an activator of the *suf* promoter after destruction of its iron-sulfur cluster by ROS (95). On the other hand, the activity of l,d-transpeptidases involves a catalytic cysteine residue that must be reduced (96), which is challenging under oxidative stress. The expression of *ldtC* from the *suf* promoter, which is strongly activated under the latter condition, is therefore biologically meaningful. Interestingly, in E. coli, the *suf* operon is also located upstream of a gene encoding an l,d-transpeptidase (*ldtA*), the two operons being also transcribed both together and separately (75).

### Concluding statement.

In this study, we combined five transcriptomic data sets yielding complementary information and designed to provide a catalogue of the genes’ responses to and RNA landscapes for various growth and stress conditions, including one of the first applications of Nanopore native RNA-seq to prokaryotic transcriptomes. Their integration through a computational method developed for this study allowed us to precisely determine and annotate the transcriptomic map of *D. dadantii*, the first of its kind in the *Dickeya* genus. The analysis of *in planta* DNA microarray data suggests that the identified TUs are also coexpressed during the early stages of plant infection, although a more refined *in planta* analysis would require higher-resolution transcriptomic data. Beyond its practical aspect as a community resource to help the scientific community unravel gene regulation, including the virulence program of this and related species, the obtained transcriptional map clearly indicates, after others, that the canonical operon model is insufficient to account for the complexity of bacterial transcription. The ability of the cell to differentially express genes of the same operon depending on metabolic needs and environmental conditions was first described with suboperonic regulation years ago. Later, with the emergence of next-generation sequencing, transcriptomic analyses confirmed at the genomic scale that most operons were able to generate alternative transcripts of variable gene composition. Transcriptional read-through at terminators is another mechanism that might play a basal coordination and regulation role and explain the extent of transcription beyond the scale of operons. Recent findings include noncontiguous operons and excludons, where the expression of an operon transcript can be mutually regulated with that of a gene located on the opposite strand at the same locus. For such features, the putative catalogue provided here may be used as a starting point for further investigation and, in particular, might be combined with the *D. dadantii* noncoding RNA landscape (97) for a comprehensive analysis of transcriptional regulation in this bacterium. Together, our findings provide insights into the mechanisms of basal coordination of transcription and might contribute to the revision of the canonical view of operon structure and transcription organization.

## MATERIALS AND METHODS

### Bacterial strain, genome annotation, and genome-wide predictions of operons.

The genome sequence and annotation files from Dickeya dadantii strain 3937 were obtained from NCBI under accession no. NC_014500.1 (98). This work focused on coding genes only (CDS; representing 4,211 genes over 4,411 in total). *D. dadantii* operons were predicted using Rockhopper, a recent computational tool for operon prediction based on RNA-seq expression data as well as genomic and functional information (40), by providing data set 1 as input.

### RNA-sequencing data (data set 1), definition of putative transcription units based on intergenic signals, and identification of unannotated genes.

Strand-specific, paired-end RNA-seq data used in this study are taken from reference 99. They comprise six *in vitro* conditions (Fig. 1, with two biological replicates each), including various growth (M63 medium supplemented with sucrose in exponential or stationary phase, in the presence or absence of PGA) and DNA supercoiling (novobiocin shock) conditions. For each sample, RNA fragments were inferred from paired-end read information, and genome-wide coverage was computed from resulting RNA fragment coordinates using a Python homemade script.

To define putative transcription units, separately for each strand, adjacent genes were fused in the same putative TU as long as the coverage was greater than 0 at each position of their intergenic region (independently of its size) for at least half of the samples (Fig. 7A).

Unannotated genes were defined as DNA regions outside of known coding sequences, longer than the first centile (1%) of *D. dadantii* gene lengths (192 bp), with an average coverage significantly different from 0 (with 99% confidence, i.e., >9 at each position) in all samples, and with a coding sequence predicted by Prodigal (100), resulting in 50 unannotated genes. A search for homolog proteins was performed using PSI-BLAST based on the nonredundant protein database (see Table S1D in the supplemental material).

### *In vitro* DNA microarray data (data set 2) and coexpression validation of the putative transcription units using hierarchical clustering.

Microarray processed data used in this study are described elsewhere (27). They comprise 32 *in vitro* conditions (with two biological replicates each), including various growth and stress conditions encountered by *D. dadantii* during plant infection (Fig. 1): cells were harvested in M63 (minimal) medium supplemented with sucrose, in exponential or stationary phase, in the presence or absence of PGA or leaf extract, and exposed or not to environmental perturbations (acidic, osmotic, and oxidative stress). Pearson’s correlation coefficients were computed among all gene pairs over all conditions on the logarithm of the normalized expression level (derived from probe intensity). For each putative TU, adjacent genes were grouped into clusters based on this correlation, using a hierarchical clustering framework constrained to group adjacent genes only, with a custom Python script. At each iteration of the algorithm, the median of cross-correlations among all clusters (or genes) was computed, and the adjacent clusters with maximal median were fused. The hierarchical clustering ends when a cutoff value, *C*, for the correlation is reached (Fig. 7B). If the agglomeration of all genes of the TU is achieved without reaching *C*, the TU is validated. Otherwise, the final clusters are considered separate TUs. A high *C* value results in short, highly correlated TUs, whereas a low *C* value yields longer, moderately correlated TUs (Table S5). We defined the value *C *= 0.25 such that 20% of operon predictions were discarded (Fig. S5A), since it is the number of false predictions (i.e., specificity) evaluated for Rockhopper in E. coli, a *D. dadantii* enterobacterium relative. Varying the precise value of *C* did not qualitatively change the main results (Table S5). The identified TUs exhibit a length distribution similar to those reported in E. coli (3, 9).

10.1128/mbio.00524-22.5 FIG S5 (A) Coexpression validation of transcription units for different correlation threshold *C* values. TUs obtained with high *C* values are more highly correlated but shorter. Putative TUs are obtained from step 1 of the analysis (intergenic signal), without any requirement for the correlation of expression. With the chosen value (*C *= 0.25), TUs group around three times more gene pairs than operons predicted by Rockhopper. The value of *C* was chosen such that 20% of operon predictions were discarded, since it is the number of false predictions of Rockhopper in E. coli, a *D. dadantii* enterobacterium relative. (B) Summary statistics of Nanopore native RNA-seq data (data set 3), including the distribution of read lengths, and mean log_2_(RPKM) across RNA-seq conditions (data set 1) depending on Nanopore read counts. The box extends from the first quartile to the third quartile values of the data, with an orange line at the median, and with whiskers extending from each end of the box to the extreme values. On average, genes with one Nanopore read count have a log_2_(RPKM) across RNA-seq conditions of 1.4. Download FIG S5, PDF file, 0.3 MB. Copyright © 2022 Forquet et al. 2022 Forquet et al. https://creativecommons.org/licenses/by/4.0/ This content is distributed under the terms of the Creative Commons Attribution 4.0 International license.

### Nanopore native RNA sequencing (data set 3), validation of the mRNA landscape, and genome-wide identification of putative transcription termination sites.

*D. dadantii* cultures were grown in M63 medium supplemented with glucose and PGA, until the early exponential phase (condition 1) or the early stationary phase (condition 2) (Fig. 1). RNAs were extracted using a frozen acid-phenol method, as previously described (101), and treated successively with Roche and BioLabs DNases. Two samples were prepared: 50 μg of RNAs from each condition was pulled into one sample (sample 1), whereas the other one contained 100 μg of RNAs from condition 2 (sample 2). Both samples were then supplied to Vertis Biotechnologie AG for Nanopore native RNA-seq: total RNA preparations were first examined by capillary electrophoresis, and rRNA molecules were depleted for sample 1 using only an in-house-developed protocol (recovery rate, 84%). RNA 3′ends were then poly(A)-tailed using poly(A) polymerase, and the Direct RNA sequencing kit (SQK-RNA002) was used to prepare the library for one-dimensional (1D) sequencing on the Oxford Nanopore sequencing device. The direct RNA libraries were sequenced on a MinION device (MIN-101B) using standard settings. Base calling of the fast5 files was performed using Guppy (version 3.6.1) with the following settings: –flowcell FLO-MIN106 –kit SQK-RNA002 –cpu_threads_per_caller 12–compress_fastq –reverse_sequence true –trim_strategy rna. Reads smaller than 50 nucleotides were removed. Raw read sequencing data are available in the EBI Gene Expression (ArrayExpress) database under accession no. E-MTAB-10482. Summary statistics are provided in Fig. S5B: 466,393 and 556,850 reads were generated from samples 1 and 2, respectively, with a median length of 358 nucleotides. Quality control was performed on both data sets using Nanopack (102), resulting in a median quality of 9.3 (88% base calling accuracy) (Fig. S5B). Long reads from the fastq files were mapped to the Dickeya dadantii strain 3937 genome (NCBI accession no. NC_014500.1) (98) using minimap2 [release minimap2-2.17 (r941)] (103). Output alignments in PAF and SAM format were generated with the recommended options for noisy Nanopore native RNA-seq, adapted to bacteria (no splicing) (-ax map-ont -k14). Secondary alignments were not reported for sample 2 due to multiple secondary alignments in rRNA region (–secondary=no). In total, 382,290 and 392,743 alignments were generated (77% and 67% mappability) from samples 1 and 2, respectively (Fig. S5B). Alignment files were further sorted, indexed, and analyzed with SAMtools. Alignments from both samples were merged into one PAF file, and the latter was used for further analyses. We found that the median Nanopore read count per gene was 30, whereas, on average, genes with one Nanopore read count have a log_2_ reads per kilobase per million (RPKM) across RNA-seq conditions of 1.4 (Fig. S5B).

For each TU previously defined with data sets 1 and 2 (Fig. 7A and B), the presence of long overlapping native RNA reads was investigated using a Python homemade script for adjacent gene pairs belonging to the same TU (Fig. 7C). If at least one RNA read overlapped the two adjacent genes, their cotranscription was validated (indicated as “validated” in Table S1A). If the signal was too weak for the investigated genes (read counts < 9, not significantly different from 0 with 99% confidence), no conclusion could be drawn (indicated as “low signal” in Table S1A). Otherwise, if no overlapping RNA was found, it was not validated (indicated as “invalidated” in Table S1A), which might also be due to the low number of conditions tested.

For the determination of TTSs, for each position of the genome, we computed the total number of RNA fragments ending at this particular position by using a Python homemade script. From this stop density, we defined putative TTSs as positions downstream of gene stop codons (up to 100 bp, based on 3′ UTR lengths in E. coli) enriched for RNA fragment stops, respectively. In each of these regions, we started from site *i* with the highest stop signal *k_i_* on 5-bp centered windows (due to the low sequencing depth). For position *i* to be considered a putative TTS, we imposed *k_i_* to be significantly different from 0 (with 95% confidence > 6). TTSs obtained with this approach are provided in Table S2D.

### Differential RNA-sequencing experiments and genome-wide identification of putative transcription start sites (data set 4).

RNAs from data set 2 (27) (*in vitro* DNA microarray data) were pooled into four samples, S1 to S4, resulting in a combination of stress (pH, NaCl, H_2_O_2_) and growth conditions: exponential phase with (S1) or without (S2) stress, transition to stationary phase with (S3) or without (S4) stress (more details are provided in Fig. 1). Those samples were then supplied to Vertis Biotechnologie AG for Terminator exonuclease (TEX) treatment and Illumina sequencing. Briefly, rRNA molecules were depleted from the total RNA samples using the Ribo-Zero rRNA removal kit for bacteria (Epicentre), and small RNAs (<200 nt) were discarded using the RNeasy MinElute cleanup kit (Qiagen). For the generation of TSS cDNA libraries, the samples were first fragmented using RNase III, poly(A)-tailed using poly(A) polymerase, and split into two halves, with one half being treated with Terminator exonuclease (+TEX; Epicentre), and the other one being left untreated (−TEX). 5′PPP structures were then converted into 5′P ends using RNA 5′ polyphosphatase (5′PP; Epicentre), to which RNA adapters were ligated. First-strand cDNAs were synthetized using an oligo(dT)-adapter primer and the Moloney murine leukemia virus (MMLV) reverse transcriptase, PCR amplified using a high-fidelity DNA polymerase, purified using the Agencourt AMPure XP kit (Beckman Coulter Genomics), and sequenced on an Illumina NextSeq 500 system (60-bp read length, single-end, strand-specific protocol). Sequencing reads were trimmed to remove poly(A) tails and adapters. The fastq sequencing files are available in the EBI Gene Expression (ArrayExpress) database under accession no. E-MTAB-9075. Putative TSS positions were then determined based on the enrichment of sequencing reads in TEX-treated samples (+TEX) compared to nontreated ones (−TEX) using TSSer, an automated annotation program, from dRNA-seq data with default parameters: TSS positions within 5 bases on the same strand were clustered together, and the position with the highest amount of read increase in the +TEX library was retained. TSSs obtained with such an approach are provided in Table S2A.

### *In planta* DNA microarray data (data set 5) and coexpression validation of the transcription units inferred from *in vitro* conditions.

Microarray processed data used in this study are described in reference 29. They comprise two conditions: bacteria were collected 6 h postinoculation of the model plant Arabidopsis thaliana by wild-type *D. dadantii* during the epiphytic colonization of the leaf surfaces (5 replicates) and 24 h postinoculation during the leaf invasion (4 replicates). Pearson’s correlation coefficient was computed among all gene pairs over the two conditions on the logarithm of the normalized expression level (derived from probe intensity) (Fig. S4).

### Genome-wide detection of transcription start and termination sites from RNA-seq data, mapping to the transcription units.

We computed the densities of RNA fragments starting and ending at each position of the genome across all RNA-seq samples (data set 1) (Fig. 7D). In order to retain only TSSs and TTSs relevant to protein coding genes, we focused on regions located upstream of gene start codons (up to 250 bp, based on 5′-UTR lengths in E. coli) and downstream of gene stop codons (up to 100 bp, based on 3′-UTR lengths in E. coli), respectively. In each of these regions, putative TSSs/TTSs were defined as sites *i* with highest start/stop signal *k_i_*. To differentiate a TSS/TTS at position *i* from the noise, we imposed two successive conditions: (i) *k_i_* is significantly different from 0 (with 99% confidence, *k_i_* > 9), and (ii) *k_i_* is greater than or equal to a density cutoff value, *D*. The latter was set as 10 times the median of the density values of the region investigated for TSSs and five times for TTSs, showing that the recorded transcripts indeed start/stop at that precise position, rather than along a poorly defined starting/stopping region. In that case, position *i* was considered a putative TSS/TTS of strength *k_i_*. Setting a low-density cutoff *D* would tend to include false positives resulting from RNA-seq signal variations (noise), whereas a high cutoff would exclude weakly expressed TSSs/TTSs. We selected the value of *D* (i) such that TSSs and TTSs were detected for known operons and experimentally characterized TUs (described in the present study) and (ii) by visually curating the density graphs and excluding many positions obviously associated with RNA-seq signal variations.

TSS/TTS positions were then compared among data sets to evaluate their confidence level. For each TSS identified with this approach, if a putative TSS obtained from data set 4 (TEX libraries) was close enough (±20 bp), its position was retained (assuming a higher precision and resolution). In addition, a scan for promoter motifs was conducted with bTSSfinder (51), which includes sequence motifs for different sigma factors inferred from an E. coli promoter database. In addition, HrpL predicted promoters were manually annotated based on previous analysis in *D. dadantii* (53) and various phytopathogenic bacteria (54). For TTSs, the same method was applied using the position of the closest predicted hairpin loop (±50 bp) or TTS positions obtained from Nanopore native RNA-seq data (data set 3). TSSs and TSSs were then assigned to the TUs, and only internal TSSs and TTSs with 15% relative amplitude (i.e., $\frac{k_{i}}{\Sigma (k_{i})}$) were retained, resulting in a total of 2,595 TSSs and 1,699 TTSs over all TUs. Setting a low relative amplitude cutoff would tend to retain all TSSs/TTSs, including many very weak ones mostly due to noise. We selected the relative amplitude cutoff value (i) based on a collection of known operons and TUs (shown in the present study) and (ii) such that the total numbers of TSSs and TTSs identified were consistent with those reported recently in E. coli (3, 75). If no TSS/TTS was found from data set 1, we indicated the closest putative one from data set 3 or 4 with a lower confidence level. The lists are provided in Table S1A to S1C.

### Detection of transcriptional readthrough at internal TTSs.

For each internal TTS, the expression ratio Δ(log_2_(RPKM)) of the gene located downstream to the gene located upstream. We imposed two successive conditions to consider the transcriptional read-through at this TTS as condition independent: (i) $\Delta ({\text{log}}_{\text{2}}(\text{RPKM}))\leq -0.5$ for at least 8 samples over 12 corresponding to at least a termination probability *P*(TTS) of 71% and (ii) a standard error of the mean σ *P*(TTS) of ≤12.5% corresponding to a relatively constant mean expression ratio and subsequent termination probability.

### Data availability.

The following data are available as indicated: (i) Dickeya dadantii strain 3937 genome sequence and annotation files under NCBI accession number NC_014500.1 (98); (ii) RNA-seq data (data set 1) under EBI Gene Expression (ArrayExpress) accession number E-MTAB-7650 (99); (iii) *in vitro* microarray data (data set 2) under EBI Gene Expression (ArrayExpress) accession number E-MTAB-541 (27); (iv) Nanopore native RNA sequencing (data set 3) under EBI Gene Expression (ArrayExpress) accession number E-MTAB-10482; (v) differential RNA-seq data (data set 4) under EBI Gene Expression (ArrayExpress) under accession number E-MTAB-9075; (vi) *in planta* microarray data (data set 5) under NCBI Gene Expression Omnibus (GEO) accession number GSE94713 (29).
