# Supplementary figures and images for: Mapping the Complex Transcriptional Landscape of the Phytopathogenic Bacterium Dickeya dadantii
Source: mBio. 2022 May 2;13(3):e00524-22. doi: 10.1128/mbio.00524-22 (PMC9239193; doi:10.1128/mbio.00524-22)

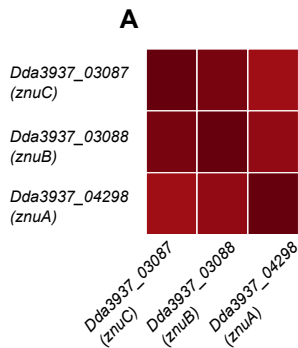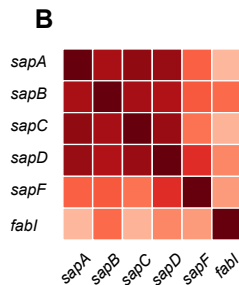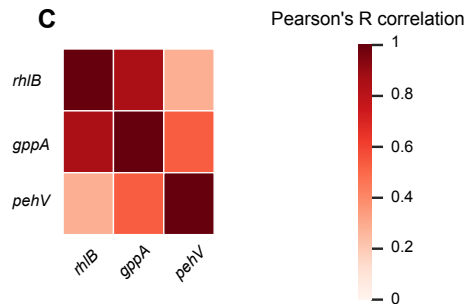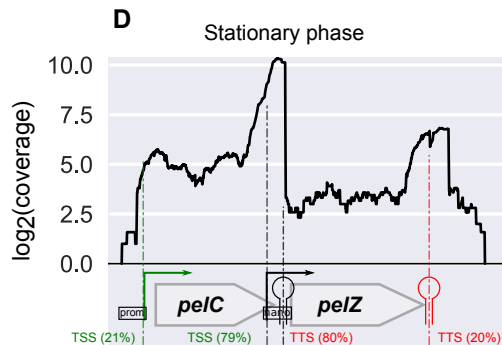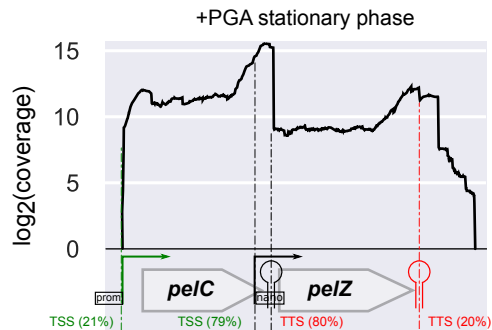

TSS (in TEX)

TSS (not in TEX)

prom predicted promoter

TTS in Nanopore

TTS (unknown type)

TTS (intrinsic)

Supplement: FIG S1 [file mbio.00524-22-sf001.pdf]

# Nanopore native RNA-seq

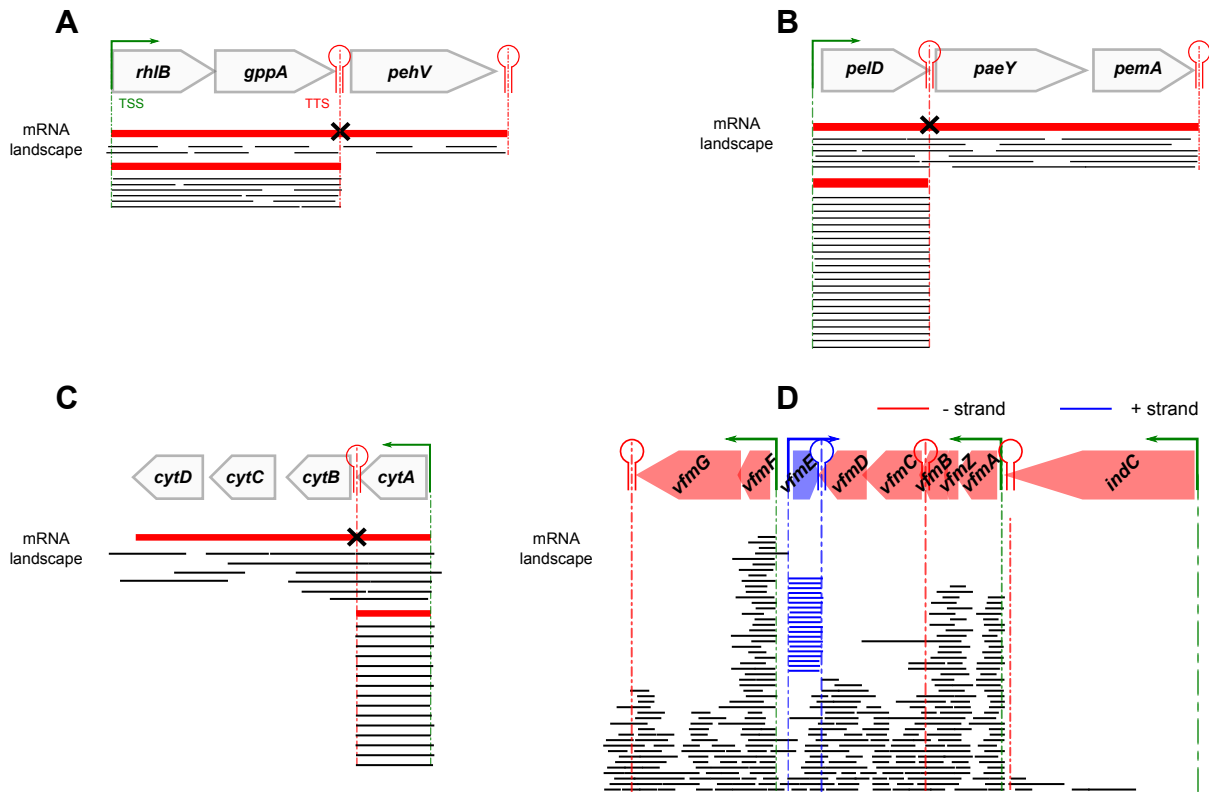

Supplement: FIG S2 [file mbio.00524-22-sf002.pdf]

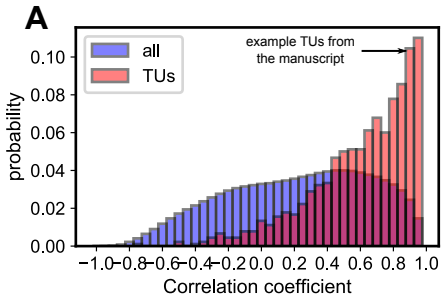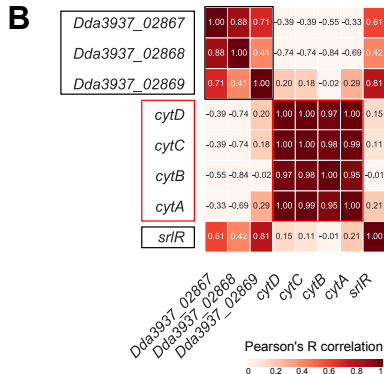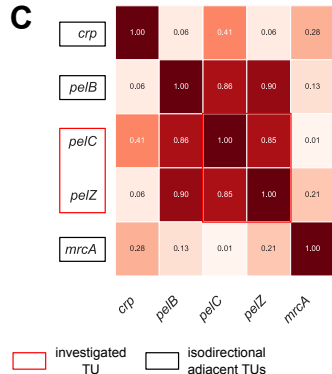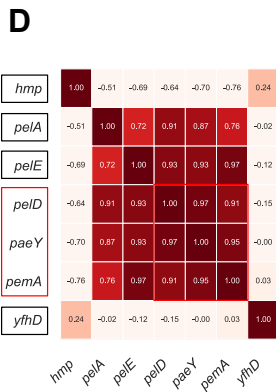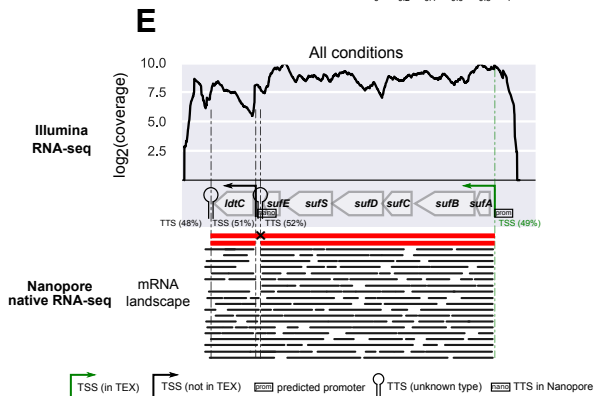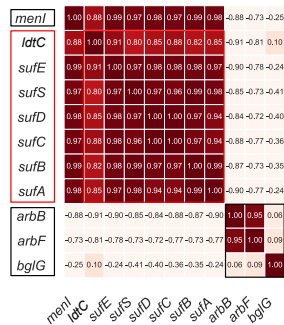

Supplement: FIG S4 [file mbio.00524-22-sf004.pdf]
