# Supplementary material for: Mapping the Complex Transcriptional Landscape of the Phytopathogenic Bacterium Dickeya dadantii
Source: mBio. 2022 May 2;13(3):e00524-22. doi: 10.1128/mbio.00524-22 (PMC9239193; doi:10.1128/mbio.00524-22)
Supplement: FIG S3 [file mbio.00524-22-sf003.pdf]

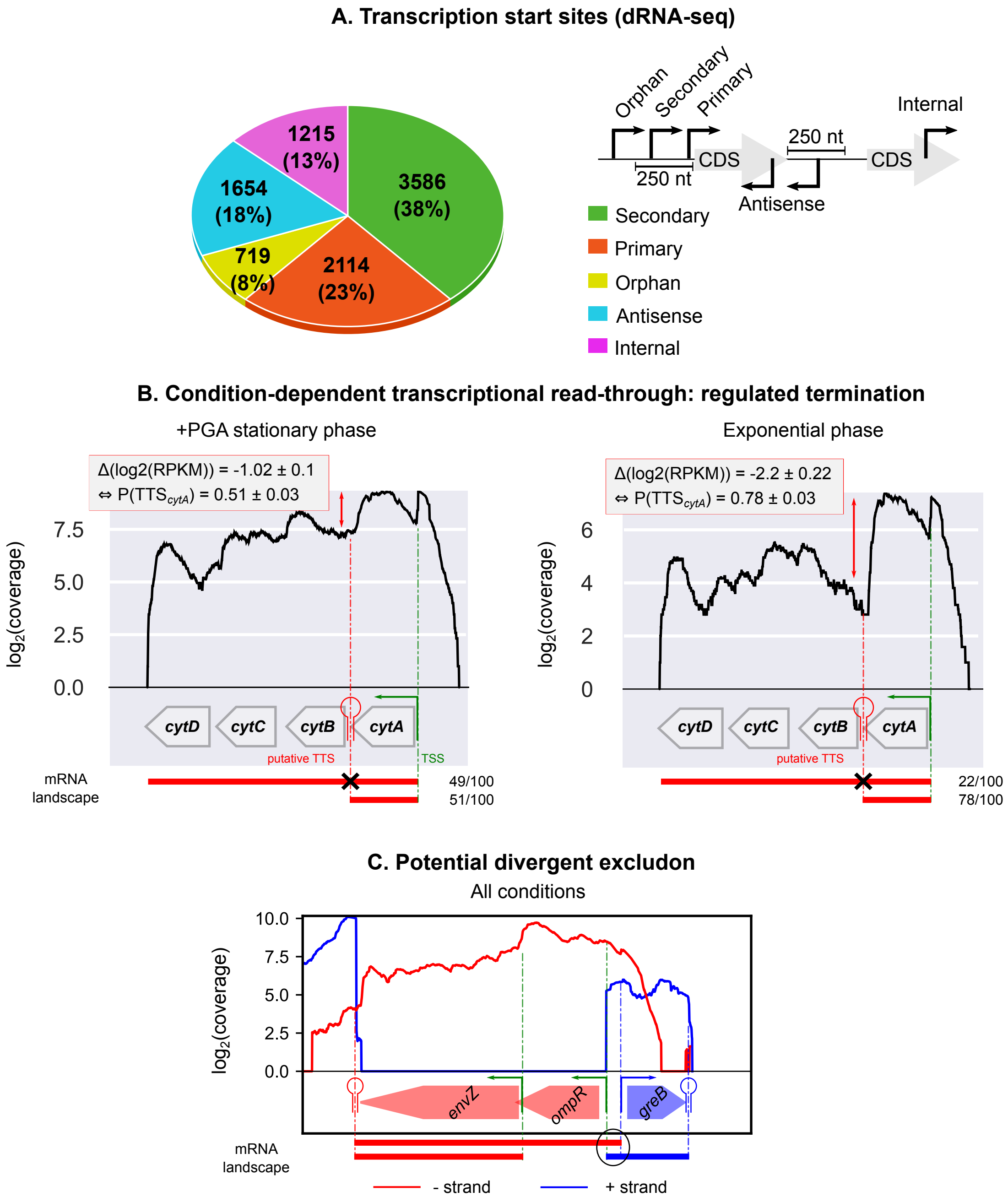

A. Transcription start sites (dRNA-seq)
1215
(13%)
1654
(18%)
3586
(38%)
719
(8%)
2114
(23%)
Orphan
Secondary
Primary
Internal
CDS
250 nt
250 nt
CDS
Antisense
Secondary
Primary
Orphan
Antisense
Internal
B. Condition-dependent transcriptional read-through: regulated termination
+PGA stationary phase
Exponential phase
Δ(log2(RPKM)) = -1.02 ± 0.1
⇔ P(TTScytA) = 0.51 ± 0.03
Δ(log2(RPKM)) = -2.2 ± 0.22
⇔ P(TTScytA) = 0.78 ± 0.03
log2(coverage)
7.5
5.0
2.5
0.0
6
4
2
0
cytD
cytC
cytB
cytA
putative TTS
TSS
mRNA
landscape
49/100
51/100
22/100
78/100
C. Potential divergent excludon
All conditions
log2(coverage)
10.0
7.5
5.0
2.5
0.0
envZ
ompR
greB
mRNA
landscape
- strand
+ strand
