# Supplementary material for: Mapping the Complex Transcriptional Landscape of the Phytopathogenic Bacterium Dickeya dadantii
Source: mBio. 2022 May 2;13(3):e00524-22. doi: 10.1128/mbio.00524-22 (PMC9239193; doi:10.1128/mbio.00524-22)

A **Co-expression validation of transcription units**

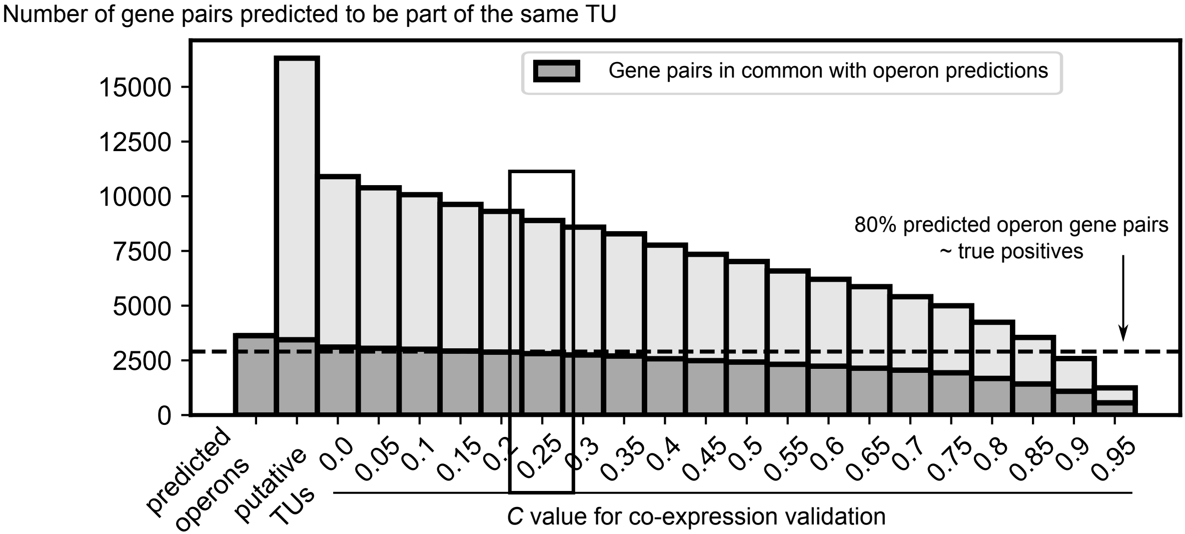

B **Summary statistics of Nanopore native RNA-seq data**

**Total reads**	466 393 (sample 1) + 556 850 (sample 2)
**Median quality**	9.3 (88% base calling accuracy)
**Median length**	358 nucleotides
**Total alignments**	382 290 + 392 743
**Genome mappability**	77 + 67 %
**Median counts/gene**	30

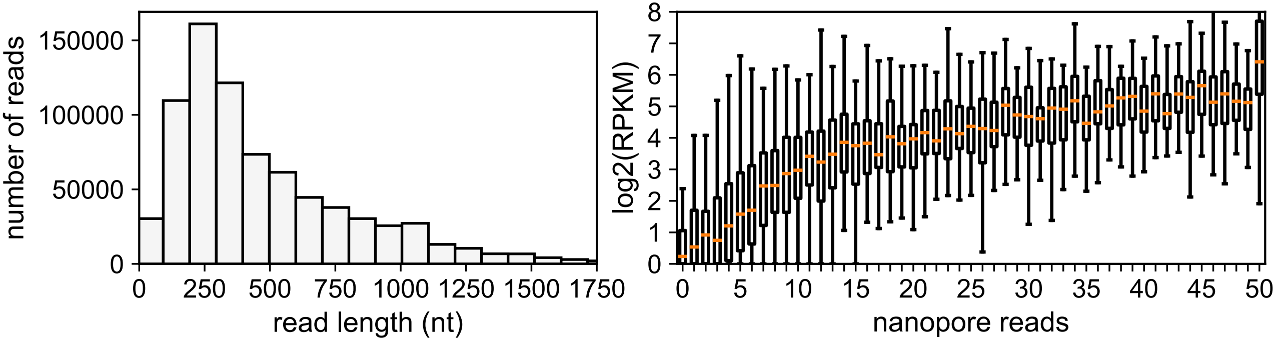

Supplement: FIG S5 [file mbio.00524-22-sf005.pdf]
